# Association between previous history of gout attack and risk of deep vein thrombosis - a nationwide population-based cohort study

**DOI:** 10.1038/srep26541

**Published:** 2016-05-27

**Authors:** Chun-Chih Chiu, Yung-Tai Chen, Chien-Yi Hsu, Chun-Chin Chang, Chin-Chou Huang, Hsin-Bang Leu, Szu-Yuan Li, Shu-Chen Kuo, Po-Hsun Huang, Jaw-Wen Chen, Shing-Jong Lin

**Affiliations:** 1 Division of Cardiology, Department of Medicine, Taipei Veterans General Hospital,Taipei, Taiwan.; 2 Cardiovascular Research Center, National Yang-Ming University,Taipei, Taiwan; 3 Division of Nephrology, Department of Medicine, Taipei Veterans General Hospital, Taipei,Taiwan; 4 Department of Medicine, Taipei City Hospital Heping Fuyou Branch, Taipei, Taiwan; 5 Institute of Clinical Medicine, National Yang-Ming University, Taipei, Taiwan; 6 Department of Medicine, Yuli branch, Taipei Veterans General Hospital, Hualien, Taiwan; 7 Department of Education, Taipei Veterans General Hospital,Taipei, Taiwan; 8 Healthcare and Management Center, Taipei Veterans General Hospital, Taipei, Taiwan; 9 National Institute of Infectious Diseases and Vaccinology, National Health Research Institutes, Miaoli County, Taiwan; 10 Department of Medical Research, Taipei Veterans General Hospital,Taipei, Taiwan; 11 Institute of Pharmacology, National Yang-Ming University,Taipei, Taiwan; 12 Taipei Medical University, Taipei, Taiwan

## Abstract

Although the association of hyperuricemia and cardiovascular diseases is well established by previous research studies, the relationship between gout and deep vein thrombosis (DVT) remains unclear. We conducted a nationwide longitudinal cohort study to investigate the relationship between gout and DVT. We used the Taiwan National Health Insurance Research Database to identify patients with gout diagnosed in Taiwan during 2000–2011, and we followed up these patients to determine the incidence of DVT among them. The association between gout and DVT was analyzed by cox proportional hazard model. The study cohort included 35,959 patients with history of gout attack and 35,959 matched controls without gout attack. During the median follow-up of 7.5 ± 3.6 years, the incidence rate of DVT was significantly higher in patients with gout than that in control group (13.48 versus 9.77 per 10^4^ person-years, p < 0.001). Kaplan-Meier analysis revealed a tendency toward DVT development in gout patients (log rank test, p < 0.001). In a Cox model, patients with gout were found to have a 1.38-fold (95% confidence interval [CI], 1.18 to 1.62, p < 0.001) higher risk of developing DVT. Hyperuricemia with gout attack could be a possible risk predictor for DVT, but these findings need to be confirmed in future clinical and experimental studies.

Venous thromboembolism (VTE), with an annual incidence of 1–3 cases per 1000 individuals, is a common cause of cardiovascular morbidity and mortality^1^. VTE constitutes a spectrum ranging from asymptomatic distal deep venous thrombosis (DVT) and sub-segmental pulmonary embolism, to limb-threatening DVT and fatal pulmonary embolism. DVT and its associated complications are sources of morbidity, including severe functional impairment due to post-thrombotic syndrome, and chronic thromboembolic pulmonary hypertension. However, DVT is often underdiagnosed, and the disease burden is underestimated^2^. Although DVT and arterial atherothrombotic disease are generally considered to be different diseases^3^, risk factors for venous and arterial thrombosis have been shown to overlap^4^. Venous thrombosis has been previously associated with red blood cells (red thrombus), and arterial thrombi are mainly composed of platelets (white thrombus). Recent epidemiological studies have suggested that patients with atherosclerosis or cardiovascular risk factors may be at increased risk of VTE^5^.

Uric acid is a byproduct of purine catabolism, of which the terminal steps are catalyzed by xanthine oxidase. High uric acid levels are often accompanied by metabolic syndrome, diabetes, hypertension, hyperlipidemia and chronic kidney disease^6^, which all contribute to the development of cardiovascular disease. Some studies have reported that hyperuricemia is considered to be associated with coronary artery disease (CAD), independently of traditional risk factors^7^,^8^,^9^, but others have argued that this association is confounded by the coexistence of cardiovascular risk factors^7^,^10^,^11^. Moreover, increased serum uric acid in humans is associated with systemic inflammation^12^, endothelial dysfunction^13^,^14^,^15^, cardiovascular disease (CVD), and cardiovascular mortality^16^,^17^. Therefore, high uric acid levels should be a link between endothelial dysfunction, pro-inflammatory, and pro-thrombotic states, and could be a possible independent risk factor for VTE. However, there are few reports on the relationship between gout attack and VTE^18^. We conducted a nationwide longitudinal cohort study to investigate the relationship between history of gout attack and DVT occurrence.

## Results

### Patient characteristics

The study cohort included 35,959 patients with history of gout attack and 35,959 matched controls without gout (Table 1). Study subjects were almost equal in gender (73.7% men), and the mean age was 54.7 years (SD = 16.1 years). The prevalence of comorbidities such as cardiovascular risk factors and traditional risks factors for DVT was similar between the gout and control groups. The median follow-up period was 7.5 ± 3.6 years for the gout group and the range was 7.4 ± 3.6 years for the control group.

### Gout attack and the risk of DVT

**Figure 1:**
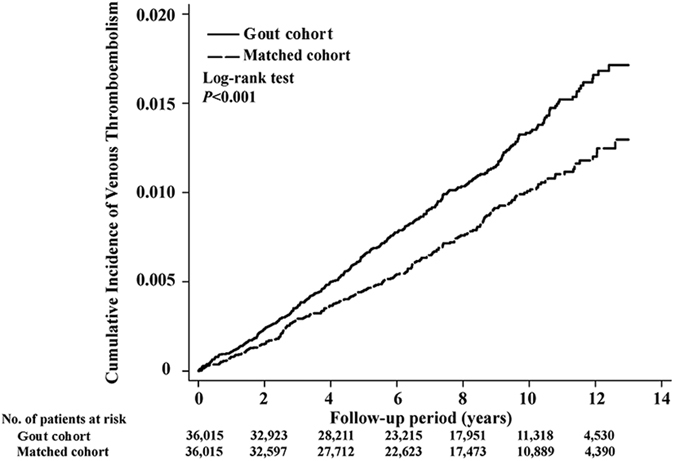


**Figure 2:**
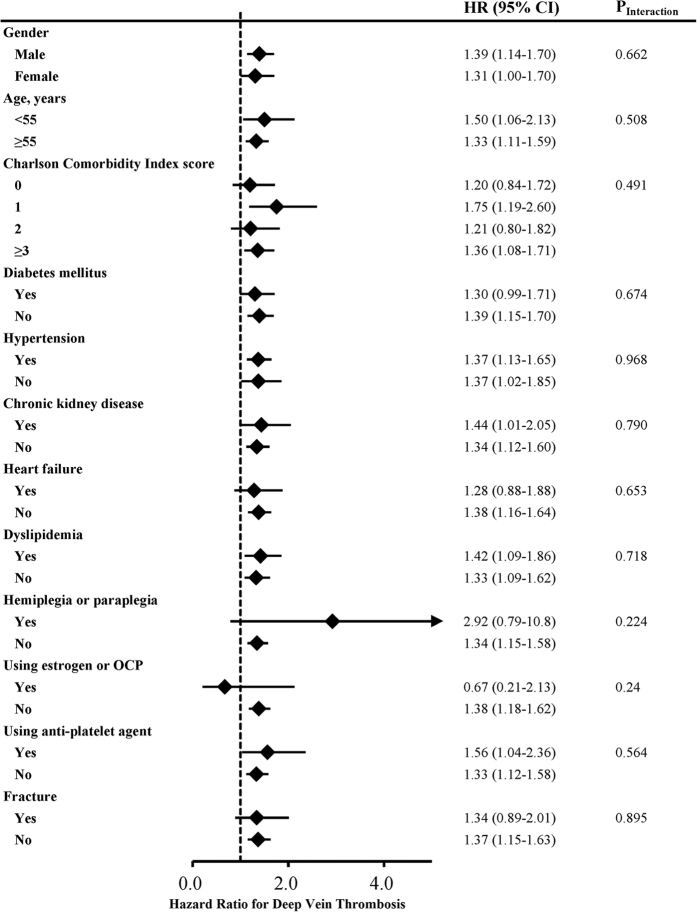


During the mean 7.4 years of the follow-up period, the incidence rate of DVT was 13.48 per 10^4^ person-years for the gout cohort and 9.77 per 10^4^ person-years for the matched control cohort (Table 2). Compared with the matched control cohort, the gout cohort had a significant risk for DVT (HR = 1.38, 95% CI, 1.18–1.62, p < 0.001). The cumulative incidence of DVT for the two groups is shown in Fig. 1. In the sensitivity analysis, after excluding traditional risk factors for DVT and other potential confounding factors, history of gout attack still significantly increased the risk of DVT (Table 3). As shown in Fig. 2, the association between gout and DVT was also consistent in subgroup analysis.

## Discussion

The role of uric acid in VTE, and particularly DVT, remains largely unknown. To the best of our knowledge, this is the first large-scale, nationwide, population-based analysis study that aimed to elucidate the relationship between gout and DVT. Our study, which included 35,959 patients with at least one episode of gout attack, demonstrated a significantly increased risk of DVT (HR = 1.38, 95% CI, 1.18–1.62, p < 0.001) after a median 7.5-year follow-up period. This association between gout and DVT was also consistent in subgroup analysis after adjustment for risk factors for atherosclerosis and thrombophilic conditions. These findings provide novel evidence that history of gout attack may be a risk predictor for DVT, and deserves a prospective study to investigate whether reducing uric acid levels could decrease DVT occurrence.

DVT is a potentially dangerous condition that can lead to considerable morbidity and severe functional impairment. Accumulating evidence has indicated that risk factors for VTE may overlap with those for arterial thrombosis. A high proportion of patients with VTE were shown to have endothelial dysfunction and an increased risk of subsequent development of cardiovascular disease^19^,^20^. Prandoni *et al*. reported an association between asymptomatic carotid artery atherosclerosis and DVT in a case-control study^21^, which suggests a close interaction between atherosclerosis and DVT. Furthermore, analysis of a secondary end-point of the JUPITER (Justification for the Use of Statins in Primary Prevention: An Intervention Trial Evaluating Rosuvastatin) trial revealed that treatment with a statin (rosuvastatin, 20 mg/day), a lipid-lowering agent, significantly reduced the risk of VTE in apparently healthy people with high levels of C-reactive protein and normal levels of low-density lipoprotein cholesterol^22^. These results suggest that atherosclerosis can induce venous thrombosis and these two conditions share common risk factors.

High uric acid concentration has been reported to be linked to endothelial dysfunction, increased free radical generation, insulin resistance and high levels of systemic inflammatory markers (such as C-reactive protein)^12^,^14^,^23^. In this nationwide cohort study, we identified the previous history of gout attack as a risk predictor of DVT occurrence, even after adjusting for traditional risk factors for cardiovascular disease and thrombophilic conditions. One particular strength of this study is the nationwide, cohort study design with age- and comorbidity-matched controls, which allows a powerful conclusion to be drawn. These findings provide clinical evidence that history of gout attack could increase DVT risk, with the potential to be a new therapeutic target for DVT prevention. However, further prospective clinical and experimental studies are needed to prove the concept.

The mechanism underlying the link of gout/hyperuricemia and DVT remains unclear. Potential mechanisms linking high uric acid levels to DVT include endothelial dysfunction, enhanced reactive oxidative stress, and pro-inflammatory and pro-thrombotic conditions in endothelial injury or dysfunction which play a critical role in VTE and atherosclerosis. Clinical studies showed that endothelial function as determined by flow-mediated dilation is inversely correlated with serum uric acid levels in subjects with asymptomatic hyperuricemia^24^,^25^. Uric acid has also been found to attenuate nitric oxide production in cultured endothelial cells, probably through induction of intracellular oxidative stress and inflammation^25^. In one retrospective study, the risk of left atrial thrombus increased in patients with non-valvular atrial fibrillation and hyperuricemia^26^. Additionally, reducing uric acid levels by administration of allopurinol, a xanthine oxidase inhibitor, improves endothelial function in different clinical conditions^27^.

Low-grade chronic inflammation, as indicated by levels of inflammatory markers such as C-reactive protein, was shown to play a critical role in atherosclerotic disease progression and VTE formation^28^. Ruggerio *et al*. reported that there is a positive and significant association between the levels of uric acid and several inflammatory markers in a large population-based sample of older persons and in a sub-sample of participants with normal uric acid concentrations^12^. Moreover, in experimental studies, uric acid stimulates the release of chemokines and synthesis of inflammatory cytokines^29^,^30^. These results strongly suggest high uric acid concentration might contribute to a pro-inflammatory state, which is an important factor for atherosclerosis progression and thrombus formation in the venous system. Our findings provide novel evidence that hyperuricemia could be associated with DVT occurrence, but further studies are warranted to clarify how uric acid causes micro-thrombus formation in the circulatory system.

There were several limitations in the present study. First, the absolute values of uric acid were not available in the nationwide dataset. However, the levels of uric acid fluctuate significantly and are not constant, which make it difficult to determine the levels appropriately. In the present study, patients were regarded as having hyperuricemia only when they had at least one episode of a gout attack requiring long-term treatment with uric acid-lowering agents. Therefore, we focused on symptomatic hyperuricemia, and the results based on this definition may be more clinically relevant. Second, diagnoses of DVT that rely on administrative claims data registered by physicians or hospitals may be less accurate than diagnoses made according to standardized criteria. All of the DVT patients in our study had received image evaluation including CT scan or Doppler sonogram, but we couldn’t obtain image report data from our database. Third, some personal information, including body mass index and smoking status, was not available in the administrative data, preventing accurate assessment of the contributory and confounding effect of these factors. Most notable among these factors is obesity, which has been reported to increase the risk of DVT^31^. Cigarette smoking has been shown, although inconsistently, to have no effect on DVT development, as reported by Ageno *et al*. in a meta-analysis^32^. In addition, the duration of hyperuricemia before study enrolled date was not obtained in our current study. Last but not the least, the propensity score matching was done for factors known at baseline and changes in risk factors over time could be a source of residual confounding.

In spite of study limitations, our findings may have significant clinical and hypothetic implications. The large-scale nationwide population-based analysis study demonstrated that patients with gout attack had a significantly increased risk of DVT after a 7.5-year follow-up period. These findings provide novel evidence that history of gout attack could be a risk predictor for DVT occurrence, which may provide some new thoughts in prevention of DVT. However, these findings would have to be confirmed in future clinical and experimental studies, including evaluation the association of inflammatory and endothelial dysfunctional markers levels and uric acid levels.

## Methods

### Data Sources

Data were extracted from the Taiwan National Health Insurance (NHI) Research Database (NHIRD), which contains anonymized secondary data that is available for research purposes. Taiwan’s NHI program, launched in 1995, currently covers 99% of the population of 23 million people. The database comprises all registry and claims data from the NHI system, ranging from demographic data to detailed orders for ambulatory and inpatient care. Taiwan’s NHI Bureau is responsible for auditing medical payments by comprehensive review of medical records, examination reports, and results of imaging studies. If physicians fail to meet the standards for clinical practice, Taiwan’s NHI reserves the right to reject payment and can impose huge financial penalties. Disease diagnoses are coded according to the International Classification of Disease, Ninth Revision, Clinical Modification (ICD-9-CM). The diagnostic accuracy for the major diseases in the NHIRD has been well validated^33^,^34^,^35^,^36^. The Longitudinal Health Insurance Database, a subset of the original NHIRD, which contains data from a random sample of 1 million NHI beneficiaries, was used in the current study.

### Study Design

This nationwide, population-based, observational, retrospective cohort study was conducted to determine the association between gout and DVT. Two cohorts were enrolled in the study: the gout cohort and a matched cohort without gout attack. The gout cohort consisted of patients with at least one episode of a gout attack necessitating long-term treatment with uric acid-lowering medications, including allopurinol and probenecid^33^. Patients with the following characteristics were excluded: age <20 years, and with history of DVT. The index date was defined as the first day of a gout attack. The control cohort comprised all patients without a diagnosis of gout. The exclusion criteria for the gout cohort were also applied to the control cohort. Index dates for subjects in the control cohort were randomly assigned and corresponded to those of patients in the gout cohort. It is a retrospective follow-up study.

We used 1:1 propensity score matching and calculated propensity scores for the likelihood of gout using baseline covariates and multivariate logistic regression analysis (Supplementary Table 1). We matched 1 control patient with each patient in the gout cohort with a similar propensity score based on nearest-neighbor matching without replacement using calipers with a width equal to 0.1 standard deviation (SD) of the log of the propensity score.

### Primary Outcome Measures

The primary outcome was hospitalization or an out-patient visit for DVT (ICD-9 453.x) and pulmonary embolism (ICD-9 415.1) with oral anticoagulant use after discharge. All of the DVT patients received image evaluation including computerized tomography (CT) scan and Doppler sonogram and were identify for the diagnosis of DVT, but we cannot confirm that all patients were submitted to these image evaluations. Both cohorts were followed until patients were diagnosed with DVT or pulmonary embolism, died, or December 31, 2012.

### Baseline Characteristics

Baseline demographic characteristics examined were age, sex, monthly income (NT$ [New Taiwan dollar] <19,100, NT$19,100–$41,999, and ≥NT$42,000), urbanization, and Charlson Comorbidity Index score. Urbanization levels in Taiwan are divided into 4 strata according to the Taiwan National Health Research Institute. Level 1 designates the most urbanized areas, and level 4 designates the least urbanized areas. The Charlson Comorbidity Index score is used to determine overall systemic health. Each increase in score indicates a stepwise increase in cumulative mortality^37^. We also identified other medications that potentially could confound the association between hyperuricemia and DVT risk.

### Statistical Analysis

Descriptive statistics were used to characterize the baseline characteristics of the study cohorts. Baseline characteristics of the 2 groups were compared by standardized mean difference. Propensity scores of the likelihood of gout were determined by multivariate logistic regression analysis, conditional on baseline covariates (Supplementary Table 1). The incidence rates of DVT in the 2 groups were calculated using Poisson distribution. The cumulative incidence or risk of DVT was estimated by use of the Kaplan-Meier method, and differences between cohorts were evaluated with the log-rank test. Cox regression models with a conditional approach using stratification were used to calculate adjusted hazard ratios (HRs) and 95% confidence intervals (CIs) for the risk of DVT^38^. We performed sensitivity analysis with the inclusion of different criteria. The likelihood ratio test was used to examine the interaction between the occurrence of DVT subsequent to gout attack and the following variables: age, sex, Charlson Comorbidity Index score, and the presence of diabetes mellitus, hypertension, chronic kidney disease, heart failure, dyslipidemia, papalysis, estrogen use, antiplatelet agent use, and fracture. Subgroup analyses were also performed.

The SQL Server 2012 (Microsoft Corp, Redmond, WA, USA) was used for data linkage, processing, and sampling. Propensity scores were calculated with SAS version 9.3 (SAS Institute, Cary, NC, USA). All other statistical analyses were conducted with STATA statistical software (version 12.0; StataCorp, College Station, TX, USA). Statistical significance was defined as *p* < 0.05.

## Additional Information

**How to cite this article**: Chiu, C.-C. *et al*. Association between previous history of gout attack and risk of deep vein thrombosis - a nationwide population-based cohort study. *Sci. Rep.* **6**, 26541; doi: 10.1038/srep26541 (2016).

## Supplementary Material

Supplementary Information

## Figures and Tables

**Table 1:** Baseline and Propensity Score–Matched Baseline Characteristics.

	**Propensity Score–Matched**		
	**Gout cohort**	**Matched cohort**	**Standardized mean difference**^**†**^
Patient No.	35,959	35,959	
Age, year (SD)	54.7 (16.1)	54.7 (16.1)	0.000
Male	26,498 (73.7)	26,500 (73.7)	0.000
Urbanization level^‡^			
1	13,273 (36.9)	13,251 (36.9)	0.001
2	20,409 (56.8)	20,424 (56.8)	−0.001
3	1,868 (5.2)	1,872 (5.2)	−0.001
4	409 (1.1)	412 (1.1)	−0.001
Monthly income			
No income	9,193 (25.6)	9,193 (25.6)	0.000
≦NT$ 19,100	6,726 (18.7)	6,719 (18.7)	0.000
NT$19,100–42,000	16,229 (45.1)	16,221 (45.1)	0.000
>NT$42,000	3,811 (10.6)	3,826 (10.6)	−0.001
Charlson Comorbidity Index score^§^			
0	10,386 (28.9)	10,410 (28.9)	−0.001
1	8,197 (22.8)	8,161 (22.7)	0.002
2	5,901 (16.4)	5,903 (16.4)	0.000
≧3	11,475 (31.9)	11,485 (31.9)	−0.001
Risk factor for DVT			
Heart failure	2,736 (7.6)	2,719 (7.6)	0.002
Hemiplegia or paraplegia	568 (1.6)	603 (1.7)	−0.008
Using estrogen or OCP	578 (1.6)	579 (1.6)	0.000
Using anti-platelet agent	3,486 (9.7)	3,482 (9.7)	0.000
Fracture	5,270 (14.7)	5,279 (14.7)	−0.001
Previous or coexisting medical condition			
Cerebrovascular disease	5,191 (14.4)	5,203 (14.5)	−0.001
Diabetes mellitus	8,236 (22.9)	8,228 (22.9)	−0.001
Hypertension	17,951 (49.9)	17,944 (49.9)	0.001
Dyslipidemia	11,701 (32.5)	11,722 (32.6)	0.000
Coronary artery disease	8,910 (24.8)	8,753 (24.3)	−0.001
Connective tissue disease	937 (2.6)	856 (2.4)	0.014
Chronic renal failure	4,421 (12.3)	4,438 (12.3)	−0.001
Chronic liver disease	9,508 (26.4)	9,516 (26.5)	−0.001
Using statin	1,423 (4.0)	1,430 (4.0)	−0.001
Using NSAID	10,484 (29.2)	10,501 (29.2)	−0.001
Using PPI	514 (1.4)	513 (1.4)	0.000
Using warfarin	145 (0.4)	172 (0.5)	−0.011
Using dipyridamole	1,669 (4.6)	1,663 (4.6)	0.001
Using anti-hyperglycemic drug	2,768 (7.7)	2,790 (7.8)	−0.002
Using anti-hypertensive drug			
Alpha-blocker	794 (2.2)	790 (2.2)	0.001
Beta-blocker	4,525 (12.6)	4,535 (12.6)	−0.001
Calcium channel blocker	6,331 (17.6)	6,343 (17.6)	−0.001
Diuretics	3,794 (10.6)	3,795 (10.6)	0.000
ACE inhibitor or ARB	4,751 (13.2)	4,742 (13.2)	0.001
Other anti-hypertensive drug	759 (2.1)	748 (2.1)	0.002
Propensity score	0.20 (0.18)	0.20 (0.18)	0.000

*All data were descripted as number (%), except mean age and propensity score.

^†^Imbalance between groups was defined as absolute value of standardized mean difference greater than 0.009. ^‡^Urbanization levels in Taiwan are divided into four strata according to the Taiwan National Health Research Institute publications. Level 1 designates the most urbanized areas, and level 4 designates the least urbanized areas. ^**§**^Charlson Comorbidity Index score is used to determine overall systemic health. With each increased level of CCI score, there are stepwise increases in the cumulative mortality. Abbreviations: SD, standard deviation; NT$, new Taiwan dollars; OCP, Oral contraceptive pill; PPI, Proton pump inhibitors; NSAIDs, Non-steroidal anti-inflammatory drugs; ACEI, angiotensin-converting-enzyme inhibitors; ARB, Angiotensin II receptor blocker.

**Table 2:** Risk of Deep Vein Thrombosis among Gout and Matched Control Cohort.

	**All patients**			**Propensity Score–Matched**	
	**No. of Event**	**Person- years**	Incidence rate*	**Hazard Ratio** (**95% CI**)	***p*** **Value**
Matched control cohort	260	266,170	9.77	As Reference	
Gout cohort	365	270,692	13.48	1.38 (1.18–1.62)	<0.001

*per 10^4^ person-years.

Abbreviations: CI, confidence interval.

**Table 3:** Sensitivity Analysis of Cox Regression Model for Risk of Deep Vein Thrombosis in Gout and Matched Control Cohorts.

	Adjusted HR* (**95% CI**)	***P*** **Value**
Primary analysis	1.38 (1.18–1.62)	<0.001
Excluding patients using anticoagulation agent	1.37 (1.16–1.60)	<0.001
Excluding patients with major surgery^†^	1.42 (1.19–1.70)	<0.001
Excluding patients with pregnancy^‡^	1.37 (1.17–1.61)	<0.001
Excluding patients using anticoagulation agent, with major surgery, and with pregnancy	1.43 (1.20–1.71)	<0.001
Excluding patients with follow-up period less than 30 days^§^	1.35 (1.15–1.58)	<0.001
Excluding patients with follow-up period less than 180 days^‖^	1.32 (1.12–1.56)	<0.001

*Adjusted for propensity score. ^†^Included all operations requiring anesthesia (general anesthesia or spinal anesthesia), and at least 1-day recumbency, was recorded within 90 days before occurrence of events or at the end of follow-up. ^‡^Included all pregnancy within 90 days before occurrence of events or at the end of follow-up. ^§^Index date was defined as the date of 31 days after enrollment to avoid immortal time bias. ^‖^Index date was defined as the date of 181 days after enrollment to avoid immortal time bias. Abbreviations: HR, hazard ratio; CI, confidence interval.
