# Supplementary material for: Association between previous history of gout attack and risk of deep vein thrombosis - a nationwide population-based cohort study
Source: Sci Rep. 2016 May 27;6:26541. doi: 10.1038/srep26541 (PMC4882589; doi:10.1038/srep26541)
Supplement: Supplementary Information [file srep26541-s1.doc]

**Association between previous history of gout attack and risk of deep vein thrombosis - a nationwide population-based cohort study**

^§^Chun-Chih Chiu^1,7^, ^§^Yung-Tai Chen^2,6,9^, Chien-Yi Hsu^1,9,10^, Chun-Chin Chang^1,9^, Chin-Chou Huang^1,4,7,8^, Hsin-Bang Leu^1,5,7,9^, Szu-Yuan Li^2,9^, Shu-Chen Kuo^9,11^,

**^*^**Po-Hsun Huang^1,7,9^, Jaw-Wen Chen^1,3,7,8^, **^*^**Shing-Jong Lin^1,3,7,9,12^

^1^Division of Cardiology, Department of Medicine, ^2^Division of Nephrology, Department of Medicine, ^3^Department of Medical Research, ^4^Department of Education, ^5^Healthcare and Management Center, Taipei Veterans General Hospital, Taipei, Taiwan; ^6^Department of Medicine, Taipei City Hospital Heping Fuyou Branch, Taipei, Taiwan; ^7^Cardiovascular Research Center, ^8^Institute of Pharmacology, ^9^Institute of Clinical Medicine, National Yang-Ming University, Taipei, Taiwan; ^10^Department of Medicine, Yuli branch, Taipei Veterans General Hospital, Hualien, Taiwan; ^11^National Institute of Infectious Diseases and Vaccinology, National Health Research Institutes, Miaoli County, Taiwan; ^12^Taipei Medical University, Taipei, Taiwan

**Running head:** gout attack and deep vein thrombosis

§ Chun-Chih Chiu and Yung-Tai Chen contributed equally to this study.

***Correspondence to Po-Hsun Huang and Shing-Jong Lin:**

Division of Cardiology, Department of Medicine, Taipei Veterans General Hospital

No. 201, Sec. 2, Shih-Pai Road, Taipei, Taiwan, R.O.C.

Tel: 886-2-2871-2121 ext. 7511

Fax: 886-2-2875-7735.

E-mail: [huangbsvgh@vghtpe.gov.tw](mailto:huangbsvgh@vghtpe.gov.tw)

| **Supplementary Table 1. Propensity Score Model Results of Probability of Diagnosis of Gout.** | | | | | |
| --- | --- | --- | --- | --- | --- |
|  |  |  | 95% CI | | |
| Parameter | Estimate | Odds Ratios | Lower | Upper | P value |
| Age, per year | 0.0185 | 1.019 | 1.018 | 1.02 | <.0001 |
| Male | 1.5997 | 4.952 | 4.817 | 5.09 | <.0001 |
| Year of Index Date |  |  |  |  |  |
| 2000 |  | 1 |  |  |  |
| 2001 | -0.0708 | 0.932 | 0.891 | 0.974 | 0.0016 |
| 2002 | -0.1251 | 0.882 | 0.843 | 0.924 | <.0001 |
| 2003 | -0.1774 | 0.837 | 0.799 | 0.877 | <.0001 |
| 2004 | -0.2537 | 0.776 | 0.74 | 0.814 | <.0001 |
| 2005 | -0.335 | 0.715 | 0.679 | 0.754 | <.0001 |
| 2006 | -0.3456 | 0.708 | 0.67 | 0.747 | <.0001 |
| 2007 | -0.3968 | 0.672 | 0.636 | 0.712 | <.0001 |
| 2008 | -0.4619 | 0.63 | 0.595 | 0.667 | <.0001 |
| 2009 | -0.5379 | 0.584 | 0.55 | 0.62 | <.0001 |
| 2010 | -0.5812 | 0.559 | 0.526 | 0.594 | <.0001 |
| 2011 | -0.6344 | 0.53 | 0.499 | 0.564 | <.0001 |
| Month of Index Date |  |  |  |  |  |
| January |  | 1 |  |  |  |
| February | -0.0117 | 0.988 | 0.93 | 1.051 | 0.7085 |
| March | -0.00227 | 0.998 | 0.942 | 1.057 | 0.9388 |
| April | 0.00674 | 1.007 | 0.951 | 1.066 | 0.8159 |
| May | -0.00525 | 0.995 | 0.941 | 1.052 | 0.853 |
| June | 0.0184 | 1.019 | 0.963 | 1.077 | 0.5191 |
| July | 0.036 | 1.037 | 0.981 | 1.096 | 0.2033 |
| August | 0.0051 | 1.005 | 0.95 | 1.063 | 0.859 |
| September | 0.0101 | 1.01 | 0.954 | 1.07 | 0.7288 |
| October | -0.0183 | 0.982 | 0.926 | 1.041 | 0.5392 |
| November | -0.0223 | 0.978 | 0.921 | 1.039 | 0.469 |
| December | -0.0241 | 0.976 | 0.918 | 1.038 | 0.4436 |
| Monthly income, NT dollars |  |  |  |  |  |
| Dependent |  | 1 |  |  |  |
| <19,100 | -0.1295 | 0.879 | 0.847 | 0.911 | <.0001 |
| 19,100−41,999 | 0.1628 | 1.177 | 1.143 | 1.212 | <.0001 |
| ≥42,000 | 0.073 | 1.076 | 1.03 | 1.123 | 0.0009 |
| Urbanization^†^ |  |  |  |  |  |
| Level 1 |  | 1 |  |  |  |
| Level 2 | 0.0289 | 1.029 | 1.004 | 1.055 | 0.0215 |
| Level 3 | 0.1169 | 1.124 | 1.064 | 1.188 | <.0001 |
| Level 4 (rural area) | 0.3652 | 1.441 | 1.287 | 1.613 | <.0001 |
| Outpatient Visits, in the past one year |  |  |  |  |  |
| 0−10 visits |  | 1 |  |  |  |
| 11−20 visits | 0.6227 | 1.864 | 1.807 | 1.923 | <.0001 |
| 21−30 visits | 0.7881 | 2.199 | 2.118 | 2.283 | <.0001 |
| 31-40 visits | 0.8408 | 2.318 | 2.212 | 2.43 | <.0001 |
| >40 visits | 0.9397 | 2.559 | 2.441 | 2.683 | <.0001 |
| Charlson Comorbidity Index Score^‡^ | -0.063 | 0.939 | 0.928 | 0.949 | <.0001 |
| Concomitant medications |  |  |  |  |  |
| Alpha-blocker | -0.1288 | 0.879 | 0.803 | 0.963 | 0.0053 |
| Beta-blocker | 0.3213 | 1.379 | 1.323 | 1.437 | <.0001 |
| Calcium channel blocker | 0.1433 | 1.154 | 1.11 | 1.2 | <.0001 |
| Diuretics | 0.8243 | 2.28 | 2.175 | 2.39 | <.0001 |
| ACE inhibitor or ARB | 0.3213 | 1.379 | 1.323 | 1.437 | <.0001 |
| Other anti-hypertensive drug | 0.7153 | 2.045 | 1.861 | 2.246 | <.0001 |
| Antiplatelet agent | -0.1134 | 0.893 | 0.849 | 0.939 | <.0001 |
| Anti-hyperglycemic drug | -0.4398 | 0.644 | 0.609 | 0.681 | <.0001 |
| Warfarin | 0.00446 | 1.004 | 0.817 | 1.236 | 0.9663 |
| Dipyridamole | 0.3879 | 1.474 | 1.38 | 1.574 | <.0001 |
| Nitrate | -0.1307 | 0.877 | 0.81 | 0.951 | 0.0014 |
| Statin | 0.00886 | 1.009 | 0.941 | 1.081 | 0.8017 |
| Using estrogen or OCP | 0.1086 | 1.115 | 1.016 | 1.223 | 0.0213 |
| SSRI | -0.3648 | 0.694 | 0.6 | 0.804 | <.0001 |
| PPI | 0.2407 | 1.272 | 1.142 | 1.417 | <.0001 |
| NSAID | 1.0428 | 2.837 | 2.76 | 2.917 | <.0001 |
| Comorbidities |  |  |  |  |  |
| Cerebrovascular disease | -0.1577 | 0.854 | 0.819 | 0.891 | <.0001 |
| Myocardial infarction | 0.000845 | 1.001 | 0.917 | 1.092 | 0.9849 |
| Hypertension | 0.751 | 2.119 | 2.051 | 2.189 | <.0001 |
| Chronic pulmonary disease | -0.00453 | 0.995 | 0.961 | 1.031 | 0.8004 |
| Diabetes mellitus | 0.1133 | 1.12 | 1.077 | 1.165 | <.0001 |
| Heart failure | 0.1823 | 1.2 | 1.135 | 1.268 | <.0001 |
| Peripheral vascular disease | 0.1201 | 1.128 | 1.045 | 1.217 | 0.0021 |
| Liver disease | 0.3195 | 1.376 | 1.333 | 1.422 | <.0001 |
| Chronic kidney disease | 0.5921 | 1.808 | 1.723 | 1.897 | <.0001 |
| Atrial fibrillation | -0.0516 | 0.95 | 0.861 | 1.047 | 0.3005 |
| Dyslipidemia | 0.8075 | 2.242 | 2.174 | 2.312 | <.0001 |
| Fracture | 0.0501 | 1.051 | 1.016 | 1.088 | 0.0044 |
| **^†^**Urbanization levels in Taiwan are divided into four strata according to the Taiwan National Health Research Institute publications. Level 1 designates the most urbanized areas, and level 4 designates the least urbanized areas. ^‡^Charlson Comorbidity Index score is used to determine overall systemic health. With each increased level of CCI score, there are stepwise increases in the cumulative mortality[.](#_ENREF_9)  Abbreviations: SD, standard deviation; NT$, new Taiwan dollars; OCP, Oral contraceptive pill; PPI, Proton pump inhibitors; NSAIDs, Non-steroidal anti-inflammatory drugs; ACEI, angiotensin-converting-enzyme inhibitors; ARB, Angiotensin II receptor blocker. | | | | | |

| **Supplementary Table 2. Subgroup Analyses of the Risk of Deep Vein Thrombosis**  **in the Gout And Matched Control Cohorts.** | | | | |
| --- | --- | --- | --- | --- |
| Characteristic | Hazard Ratio  (95% CI) | *P* value | | Interaction  *P* value |
| Gender |  |  |  | |
| Male | 1.394 (1.142-1.701) | 0.001 | | 0.662 |
| Female | 1.307 (1.002-1.704) | 0.048 | |  |
| Age, years |  |  |  | |
| <55 | 1.501 (1.058-2.128) | 0.023 | | 0.508 |
| ≥55 | 1.328 (1.111-1.588) | 0.002 | |  |
| Charlson Comorbidity Index score |  |  |  | |
| 0 | 1.199 (0.837-1.718) | 0.323 | | 0.491 |
| 1 | 1.754 (1.185-2.598) | 0.005 | |  |
| 2 | 1.209 (0.802-1.820) | 0.364 | |  |
| ≥3 | 1.357 (1.080-1.705) | 0.009 | |  |
| Diabetes mellitus |  |  |  | |
| Yes | 1.302 (0.989-1.714) | 0.060 | | 0.674 |
| No | 1.394 (1.147-1.695) | 0.001 | |  |
| Hypertension |  |  |  | |
| Yes | 1.368 (1.133-1.649) | 0.001 | | 0.968 |
| No | 1.372 (1.016-1.853) | 0.039 | |  |
| Chronic kidney disease |  |  |  | |
| Yes | 1.436 (1.005-2.052) | 0.047 | | 0.790 |
| No | 1.342 (1.123-1.603) | 0.001 | |  |
| Heart failure |  |  |  | |
| Yes | 1.282 (0.875-1.879) | 0.203 | | 0.653 |
| No | 1.379 (1.158-1.643) | <0.001 | |  |
| Dyslipidemia |  |  |  | |
| Yes | 1.419 (1.085-1.855) | 0.011 | | 0.718 |
| No | 1.328 (1.090-1.618) | 0.005 | |  |
| Hemiplegia or paraplegia |  |  |  | |
| Yes | 2.923 (0.791-10.809) | 0.108 | | 0.224 |
| No | 1.344 (1.145-1.578) | <0.001 | |  |
| Using estrogen or OCP |  |  |  | |
| Yes | 0.671 (0.211-2.129) | 0.498 | | 0.240 |
| No | 1.380 (1.175-1.621) | <0.001 | |  |
| Using anti-platelet agent |  |  |  | |
| Yes | 1.564 (1.036-2.360) | 0.033 | | 0.564 |
| No | 1.333 (1.122-1.584) | 0.001 | |  |
| Fracture |  |  |  | |
| Yes | 1.338 (0.891-2.008) | 0.160 | | 0.895 |
| No | 1.368 (1.151-1.627) | <0.001 | |  |
| * ACEI cohort as reference group  *Abbreviations*: CI, confidence interval; OCP, Oral contraceptive pill. | | | | |
